# Author Correction: A Bayesian decision support system for automated insulin doses in adults with type 1 diabetes on multiple daily injections: a randomized controlled trial

**DOI:** 10.1038/s41467-026-72173-6

**Published:** 2026-04-17

**Authors:** Alessandra Kobayati, Anas El Fathi, Natasha Garfield, Laurent Legault, Adnan Jafar, Jean-François Yale, Michael A. Tsoukas, Ahmad Haidar

**Affiliations:** 1 Division of Experimental Medicine, Department of Medicine, McGill University, Montreal, QC Canada; 2 Research Institute of the McGill University Health Center, McGill University, Montreal, QC Canada; 3 Department of Biomedical Engineering, McGill University, Montreal, QC Canada; 4 Center for Diabetes Technology, University of Virginia, Charlottesville, VA US; 5 Division of Endocrinology, Department of Medicine, McGill University Health Center, Montreal, QC Canada; 6 Department of Pediatrics, Montreal Children’s Hospital, Montreal, QC Canada

Correction to: *Nature Communications* 10.1038/s41467-025-63671-0, published online 29 September 2025

In the version of the article initially published, exploratory results from a modified version of the Diabetes Treatment Satisfaction Questionnaire (DTSQ) were reported but have now been removed from the HTML and PDF versions of the article.

