# Supplementary material for: A Bayesian decision support system for automated insulin doses in adults with type 1 diabetes on multiple daily injections: a randomized controlled trial
Source: Nat Commun. 2025 Sep 29;16:8593. doi: 10.1038/s41467-025-63671-0 (PMC12479799; doi:10.1038/s41467-025-63671-0)
Supplement: Supplementary file 1 — Supplementary Information [file 41467_2025_63671_MOESM1_ESM.pdf]

Supplementary Table 1 | Daily user interactions with main app features over 12 weeks.

	Experimental arm (n=42)	Control arm (n=42)	p value
Meal bolus (/day)	2.7 [2.4–3.1]	2.5 [1.9–3.0]	0.11
Correction bolus (/day)	0.56 [0.17–1.3]	0.46 [0.21–1.2]	0.93
Basal log (/day)	0.92 [0.82–0.98]	0.86 [0.67–0.94]	0.0078

Data are presented as median [IQR]. A two-sided Wilcoxon rank-sum test was used to compare outcomes between groups. No adjustments were made for multiple comparisons.

Supplementary Table 2 | Overall (24-hour), daytime (07:00-23:00), and nighttime (23:00-07:00) profiles for CGM and insulin outcomes.

	Experimental arm (n=42)	Control arm (n=42)	p value
Overall (24-hour)			
Percent time 3.9–10.0 mmol/L	48 (12)	45 (14)	0.47
Percent time 3.9–7.8 mmol/L	29 (9.4)	27 (11)	0.27
Percent time <3.9 mmol/L	5.9 [3.2–9.2]	4.3 [2.2–7.7]	0.16
Percent time <3.0 mmol/L	1.7 [0.67–3.4]	1.2 [0.57–2.8]	0.33
Percent time >7.8 mmol/L	65 (12)	69 (13)	0.22
Percent time >10.0 mmol/L	46 (14)	50 (16)	0.36
Percent time >13.9 mmol/L	18 [11–26]	20 [12–29]	0.57
Mean glucose (mmol/L)	10.0 [8.8–11]	10.5 [9.1–11]	0.33
SD glucose (mmol/L)	4.2 (0.78)	4.1 (0.80)	0.72
Total Insulin (U/day)	50.7 (19.0)	49.8 (21.6)	0.85
Basal Insulin (U/day)	26.1 (12.3)	24.9 (11.1)	0.65
Bolus Insulin (U/day)	24.6 (10.3)	25.0 (13.6)	0.87
Daytime (07:00-23:00)			
Percent time 3.9–10.0 mmol/L	48 (13)	46 (15)	0.68
Percent time 3.9–7.8 mmol/L	29 (9.6)	28 (11)	0.48
Percent time <3.9 mmol/L	4.5 [1.8–7.0]	3.1 [1.6–7.2]	0.50
Percent time <3.0 mmol/L	1.1 [0.25–2.5]	0.68 [0.22–2.4]	0.55
Percent time >7.8 mmol/L	67 (12)	69 (14)	0.46
Percent time >10.0 mmol/L	48 (15)	49 (17)	0.63
Percent time >13.9 mmol/L	21 [12–27]	21 [11–30]	0.82
Mean glucose (mmol/L)	10.1 [9.1–11]	10.5 [8.9–12]	0.67
SD glucose (mmol/L)	4.2 (0.77)	4.1 (0.85)	0.67
Nighttime (23:00-07:00)			
Percent time 3.9–10.0 mmol/L	47 (14)	44 (14)	0.25
Percent time 3.9–7.8 mmol/L	30 [24–37]	25 [19–30]	0.067
Percent time <3.9 mmol/L	6.9 [4.2–11]	5.1 [2.1–8.9]	0.13
Percent time <3.0 mmol/L	2.2 [1.4–5.0]	1.8 [0.47–4.1]	0.31
Percent time >7.8 mmol/L	63 (15)	69 (15)	0.089
Percent time >10.0 mmol/L	45 (17)	50 (17)	0.15
Percent time >13.9 mmol/L	18 [9.4–24]	19 [12–25]	0.38
Mean glucose (mmol/L)	9.8 [8.4–11]	10.2 [9.3–11]	0.13
SD glucose (mmol/L)	4.0 [3.7–4.5]	4.0 [3.7–4.4]	0.90

Data are presented as mean (SD) or median [IQR]. A two-sided unpaired t-test was used for normally distributed data, and a two-sided Wilcoxon rank-sum test was used for non-normally distributed data. No adjustments were made for multiple comparisons.

Supplementary Table 3 | Changes in insulin dosing parameters from baseline to Week 12.

	Experimental arm (n=42)	Control arm (n=42)	p value
Basal insulin (U/day)	0.5 (7.0)	0.2 (1.2)	0.80
Prandial insulin (%)	0.8 [-14.4–38.8]	0.0 [0.0–0.0]	0.27

Data are presented as mean (SD) or median [IQR]. A two-sided unpaired t-test was used to compare outcomes between groups. No adjustments were made for multiple comparisons.

Supplementary Table 4 | Absolute relative change (%) in prandial bolus and basal insulin dose parameters from baseline to Week 12.

	Experimental, n (%)		Control, n (%)	
Proportion of participants with changes:	Basal dose	Bolus dose*	Basal dose	Bolus dose*
>|10%|	25 (60)	29 (69)	4 (9.5)	7 (17)
>|20%|	15 (36)	19 (45)	2 (4.8)	3 (7.1)
>|30%|	9 (21)	13 (31)	2 (4.8)	1 (2.4)
>|40%|	1 (2.4)	10 (24)	1 (2.4)	0 (0)
>|50%|	0 (0)	5 (12)	0 (0)	0 (0)
>|60%|	0 (0)	4 (9.5)	0 (0)	0 (0)
>|70%|	0 (0)	3 (7.1)	0 (0)	0 (0)
>|80%|	0 (0)	1 (2.4)	0 (0)	0 (0)
>|90%|	0 (0)	1 (2.4)	0 (0)	0 (0)
>|100%|	0 (0)	1 (2.4)	0 (0)	0 (0)

*Bolus dose includes combined mean relative changes for breakfast, lunch, and dinner.

Supplementary Table 5 | Proportion of experimental participants with adjusted basal and prandial bolus insulin combinations from Week 12 compared to baseline.

	Proportion of experimental participants, n (%)
Basal and bolus insulin increased	12 (29)
Basal and bolus insulin decreased	11 (26)
Basal increased and bolus decreased	9 (21)
Basal decreased and bolus increased	10 (24)

Supplementary Table 6 | Thematic analysis of interviews with 24 sub-study participants.

Themes	Sub-themes	Selected quotes
1) Enhanced glycemia due to personalized dose recommend-ations	1.1) Streamlined access to timely decision support	*"The 6-month patient visit in the hospital, um, to let that go for such a long period of time could have negative consequences to one's health. This [algorithm], at least gives, you know, a guidance immediately in real-time, and again, it does give the patient the option to override." (P116, experimental)*<br><br>*"The adjustments made weekly was extremely helpful versus having to wait for your 4-month or 6-month meeting with a professional… changing the doses is something we rely on medical professionals to do, um, I've worked on changing them by myself at times, and there's always that fear, "have I gone too far"…if the application is as safe as professionals, then it's like having that contact but on a regular basis." (P119, experimental)*<br><br>*"My ratios have changed in the past few months, and they haven't changed in 10 plus years. So I'm basing that off of there's been more data, more information given, so I think it's definitely beneficial…For the first time in years, I feel like [pause] I don't know how to explain it, but that I have direct feedback… It's a no brainer." (P124, experimental)*<br><br>*"It's like having a real doctor with you 100% making adjustments every week...now in 2020, you need to have this in your phone, that makes it a lot better." (P122, experimental).*
	1.2) Appreciation for conservative (gradual and incremental) algorithm adjustments	*"The little changes made a difference in the long run, so for the week that a dose would increase by one unit…you do see that even if changes that were made seem insignificant, they were significant enough to make a difference." (P119, experimental)*<br><br>*"When I see the numbers come in, they're very minimal in terms of change…but it ended up working out" (P124, experimental)*<br><br>*"It's not a crazy jump week over week when there are changes…I was happy to see that…sometimes there's only one adjustment… it's gradual, it slowly adjusts to the required dosage" (P126, experimental)*
	1.3) Trust in technology	*"I had complete confidence in the recommendations, it was going really well, you could really see that there was a good change." (P115, experimental)*<br><br>*"I didn't pay attention to the recommendations, I trusted it 100%, I was very satisfied, and I'm sure that if I had paid attention, they would have been really good recommendations, but I had 100% confidence." (P122, experimental)*<br><br>*"The fast acting insulin changes, I 100% trust those…I would say, overall, very trustworthy" (P124, experimental)*

		"After a week I was pretty comfortable with it…I was giving [insulin] and I wasn't going low or anything…until basically you understand what the app is trying to do, and, uh basically, seeing that your sugar is in a normal range, you do get a trust…I would say it's easier as time progresses." (P126, experimental)
2) Advantage of digital solutions over traditional standard of care	2.1) Enhanced dosing regimen from real-time insulin-on-board tracking and insulin delivery history	*"I like the fact that it [the app] kept track of how much insulin I had in my body at the time, so it won't overdose me, which I can't do on my own" (P017, control)*<br><br>*"I could check for example the number of units that I had given myself the previous meal, which normally, I can't do…I can't remember what I gave myself, whereas here, I could check it if I wanted to. That's perhaps the most striking difference." (P018, control)*<br><br>*"What I liked the most was being able to know how many units of insulin were onboarding, so you know, not to like stack my insulin doses, that, by far was my favorite thing of the entire app." (P025, control)*<br><br>*"In the past, if I was high, I would take insulin over and over again, you know, over the period of half an hour realizing it's not changing, whereas now I just let it wait and it does come back down eventually…it tells me not to and basically just to be patient" (P124, experimental)*
	2.2) Experiential learning	*"There were a few times where I would think, "do I need to correct [with insulin for carb intake] or not", and if the calculated [insulin] dose [by the app] was zero, I wouldn't log it [the carbs]…I think for me, it was more of–or for anybody who's just checking on the basis of, "can I eat right now?" could be used in that sense" (P119, experimental)*<br><br>*"I could learn to play the doctor, I could see what's going on at the back. Like I could see that because I did this, because I got that…I can see how effective it is…so it was great to see that at the same time" (P120, experimental)*<br><br>*"I was using the app in ways where if I had a low blood sugar, I would kind of see where I can get some free carbs…putting in 15 or putting in 20 grams and seeing how high [in carbs] I can go before it tells me I need one unit [of insulin]." (P124, experimental)*
	2.3) Alleviation of mental burden associated with dose calculations	*"I don't even have to think about it, I just have to enter, I don't have to do any mental math…it takes most of the guesswork out of taking insulin…the iBolus app did the rest of the work for me, so it was very convenient." (P026, control)*<br><br>*"…it calculated my insulin for me…I've been diabetic since I was 13 and I still can't stand doing the mental math 'cause it's just like–I just prefer that it does it for me." (P117, experimental)*

	2.4) User-friendliness	*"Easy to use, very visual. There were really only three dropdown menus, so very easy to follow...everything was necessary and easy to find" (P014, control)*<br><br>*"I liked how easy it [iBolus app] is…what I need was there and easy to find, um, the interface itself was, like, perfect…there's no extra gibberish in it, you know, so I think that for any age group it's very straightforward and easy to use." (P025, control)*<br><br>*"Very easy to use, simple, very concise, um, the information required is very clear. In terms of entering the data, notes, that was all–it was an easy interface, so user friendly and easy to understand as well." (P119, experimental)*
3) Desire for advanced MDI technologies in practice	3.1) Anticipated future adoption	*"I'd opt for it again… it helps you to have a better, uh, better follow-up of your diabetes, it helps you to improve…To have it directly in the phone, it's wonderful, you know every week, it's really better than waiting 3 to 6 months." (P115, experimental)*<br><br>*"Sign me up for a rerun [laughing], I'm kind of sad of having to stop the treatment simply because it has been working well, so, um, I–I do see where potentially this could be life-changing for people, and I would like to see it continue forward…I absolutely think I would be gung ho for it." (P119, experimental)*<br><br>*"It's way, way, much better just receiving the adjustment on the phone, uh, on the app. Oh yeah, it's very convenient…And if we could have the doctor also get involved and see the results, that would be a great advantage" (P120, experimental)*<br><br>*"it's the new generation of diabetes apps. I think it would be a plus for everyone to have it because it definitely, uh, adjusts in real time…I'd definitely use it, that's for sure because I find–in fact, it makes my life a lot easier. It was more efficient" (P122, experimental)*

Supplementary Table 7 | Exit interview topic guide.

**Initial questions**	• Can you describe what your overall experience using multiple daily injections has been like throughout your participation in the study, in comparison to your usual management before you participated?
**More specific questions**	• Can you tell me about your experience using Freestyle Libre sensors throughout the study, in terms of convenience?<br>• Can you describe your overall experience using the iBolus app?<br>o What specific features did you like the most from the iBolus app?<br>o What specific features did you like the least from the iBolus app?<br>o What features, if any, would you have liked to see?<br>• If you could, would you incorporate the iBolus app into your daily insulin management?<br>• How have your hypoglycemia concerns or fears changed since before the intervention began?<br>• How have your hyperglycemia concerns or fears changed since before the intervention began?<br>• Can you tell me about the personalized recommendations that you received, in terms of your overall treatment satisfaction?<br>o What about in terms of your overall glucose control?<br>o Can you describe your overall level of trust with the recommendations that you received?<br>o What did you think about the weekly frequency in which you received the recommendations?<br>o What are your thoughts on receiving insulin dose adjustments directly from the iBolus app, compared to traditional in-hospital follow-up visits?<br>o What were the advantages of receiving personalized recommendations?<br>o What were the disadvantages?
**Final questions**	• If the learning algorithm was integrated into the iBolus app, which would allow for regular dose adjustments, would you switch to this advanced form of therapy, and why?<br>• Is there anything else that you would like to tell us about your experience with the software used in the study?

Topic guide represents the general flow used to frame interviews; questions in red were asked only to the experimental participants.

Supplementary Table 8 | Comparison of outcomes between concomitant real-time and intermittently scanned CGM use in 8 sub-study participants.

	Intermittently scanned CGM	Real-time CGM	p value
Hypoglycemic events, n	10 (9.8)	3.0 (2.6)	0.044
Percent time 3.9–10 mmol/L	47 (12)	41 (18)	0.083
Percent time <3.9 mmol/L	4.7 [1.4–7.7]	1.1 [0.40–2.6]	0.016
Percent time <3.0 mmol/L	1.5 [0.17–2.1]	0.10 [0.050–0.28]	0.016
Percent time >10 mmol/L	47 (16)	57 (20)	0.010
Percent time >13.9 mmol/L	18 (10)	26 (15)	0.053
Percent time >16.7 mmol/L	6.3 (6.6)	8.4 (9.4)	0.15
Mean glucose (mmol/L)	10.0 (1.6)	11.0 (1.9)	0.041

Data are presented as mean (SD) or median [IQR]. A two-sided unpaired t-test was used for normally distributed data, and a two-sided Wilcoxon rank-sum test was used for non-normally distributed data. No adjustments were made for multiple comparisons.

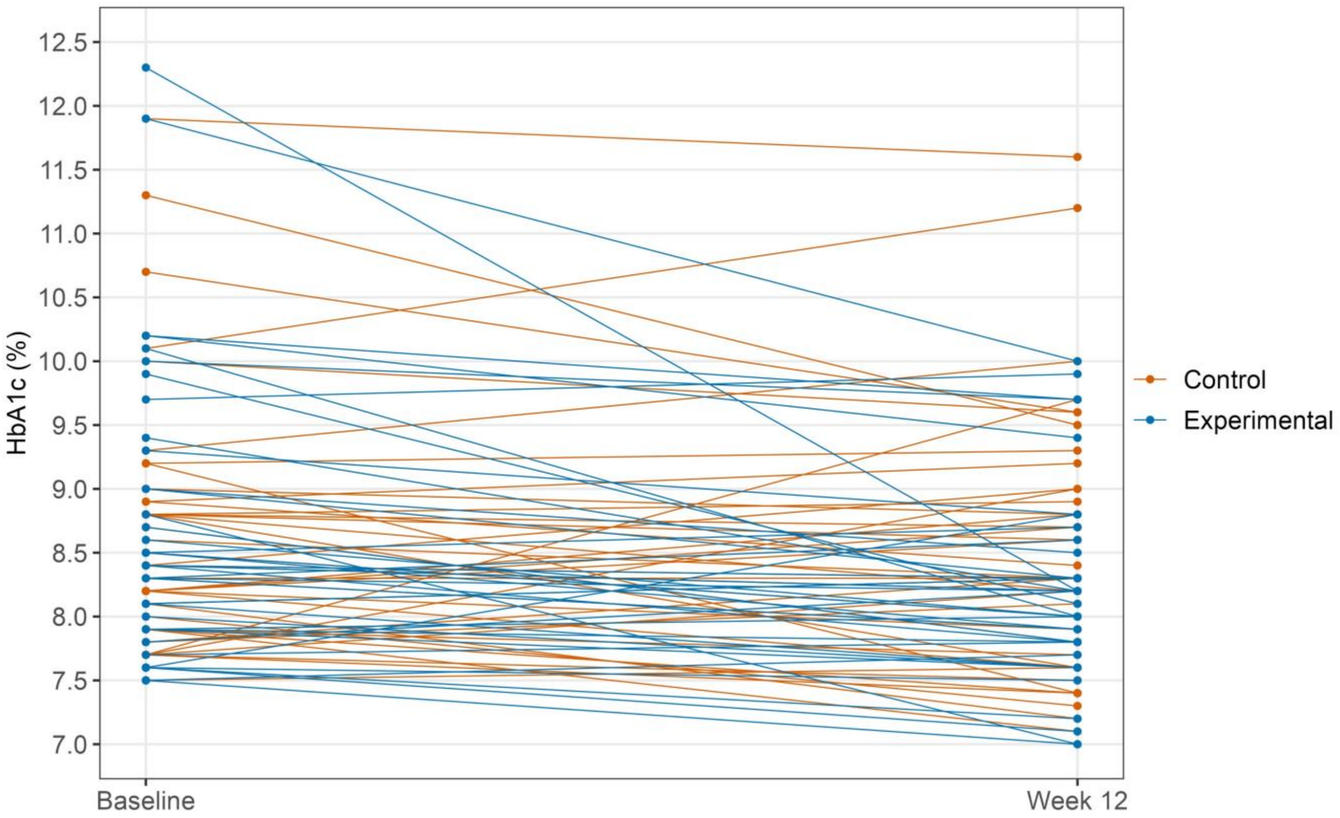

Supplementary Fig. 1 | Individual change in HbA1c from baseline to Week 12.
Individual participant changes in HbA1c (%) from baseline to Week 12 in the experimental (n=42; blue) and control (n=42; orange) arms. Each line represents one participant.

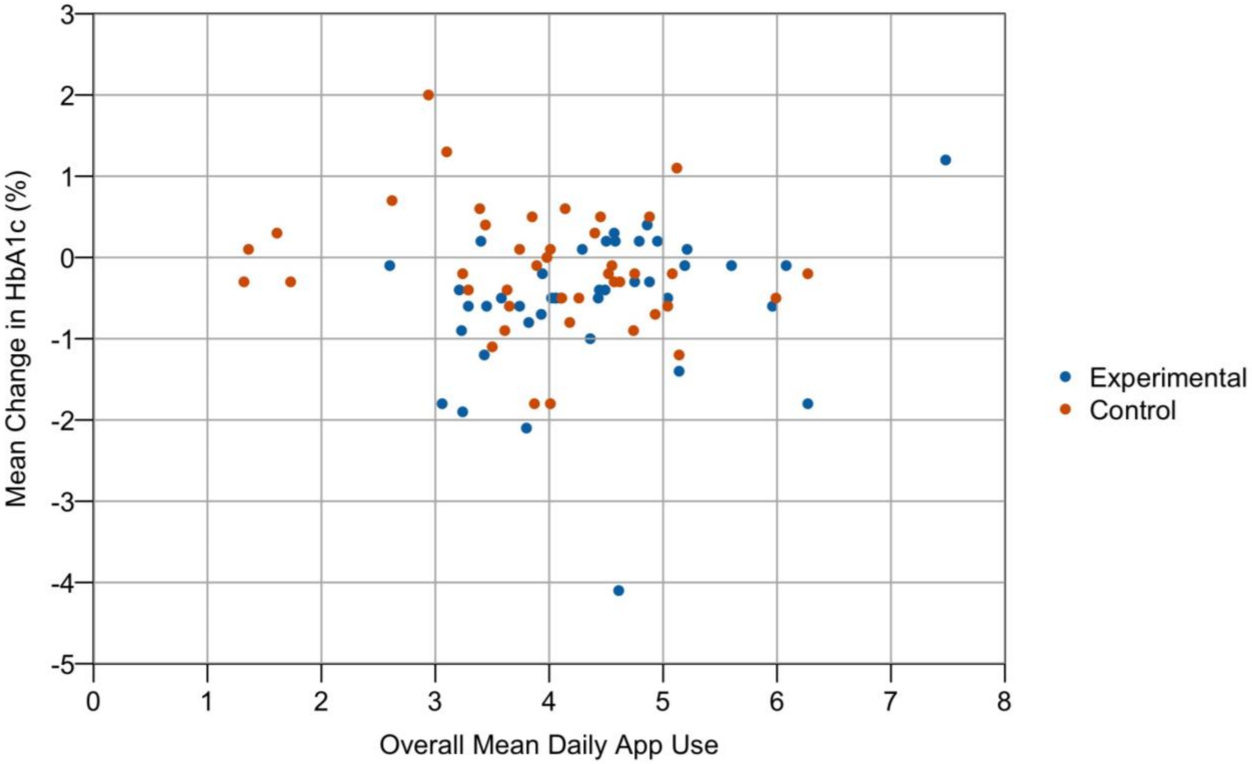

Supplementary Fig. 2 | Relationship between app use and change in HbA1c.
Scatter plot showing the association between the overall mean daily app use (x-axis) and mean change in HbA1c (%) from baseline to Week 12 (y-axis) for experimental (blue) and control (orange) participants. Each point represents one participant.

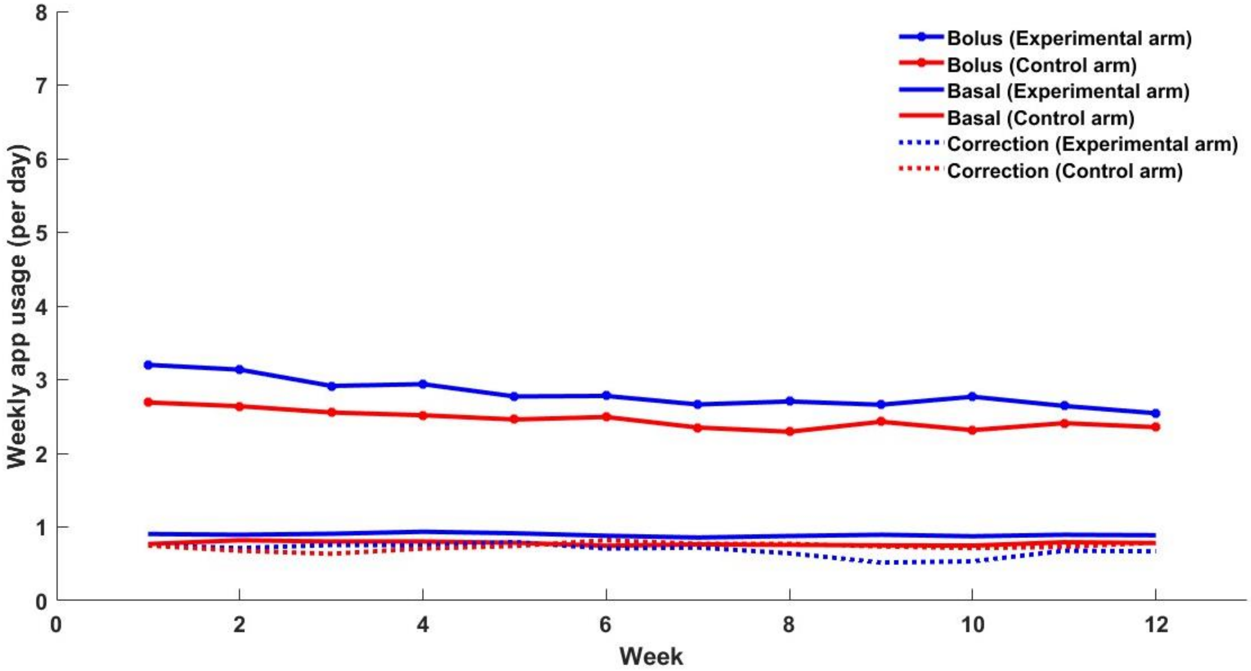

Supplementary Fig. 3 | Weekly app usage by study arm.
Week-by-week average app usage per day in experimental (n=42; blue) and control (n=42; red) participants over 12 weeks. Large-dotted lines represent use of the meal bolus feature. Solid lines represent use of basal log feature. Small-dotted lines represent use of correction bolus feature.

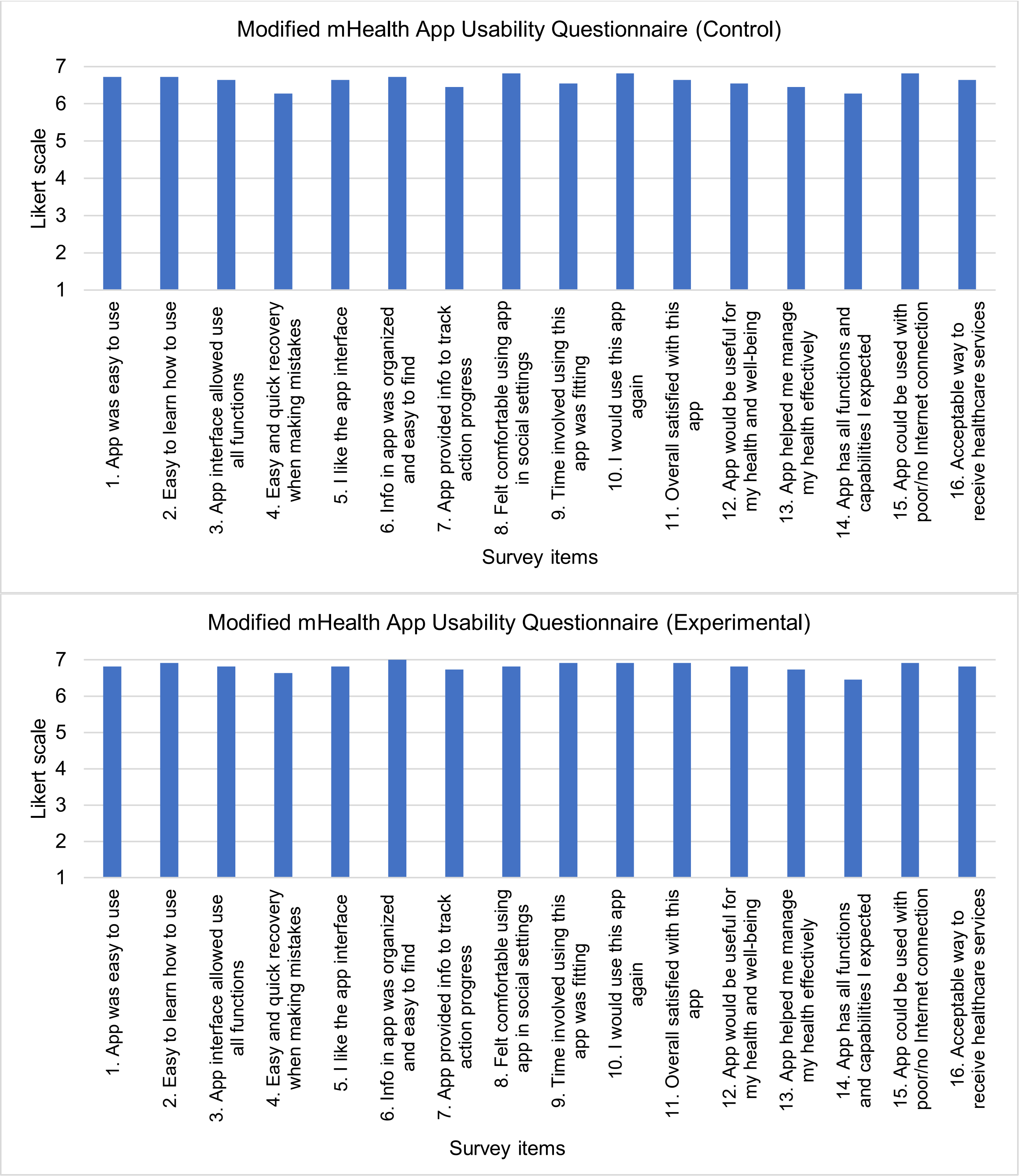

Supplementary Fig. 4 | Modified mHealth App Usability Questionnaire scores.
Scores from modified mHealth App Usability Questionnaire at end-of-study for control (n=11; top bars) and experimental (n=11; bottom bars) sub-study participants. Survey items were rated on a 7-point Likert scale (1 = strongly disagree to 7 = strongly agree). Bars represent mean scores.

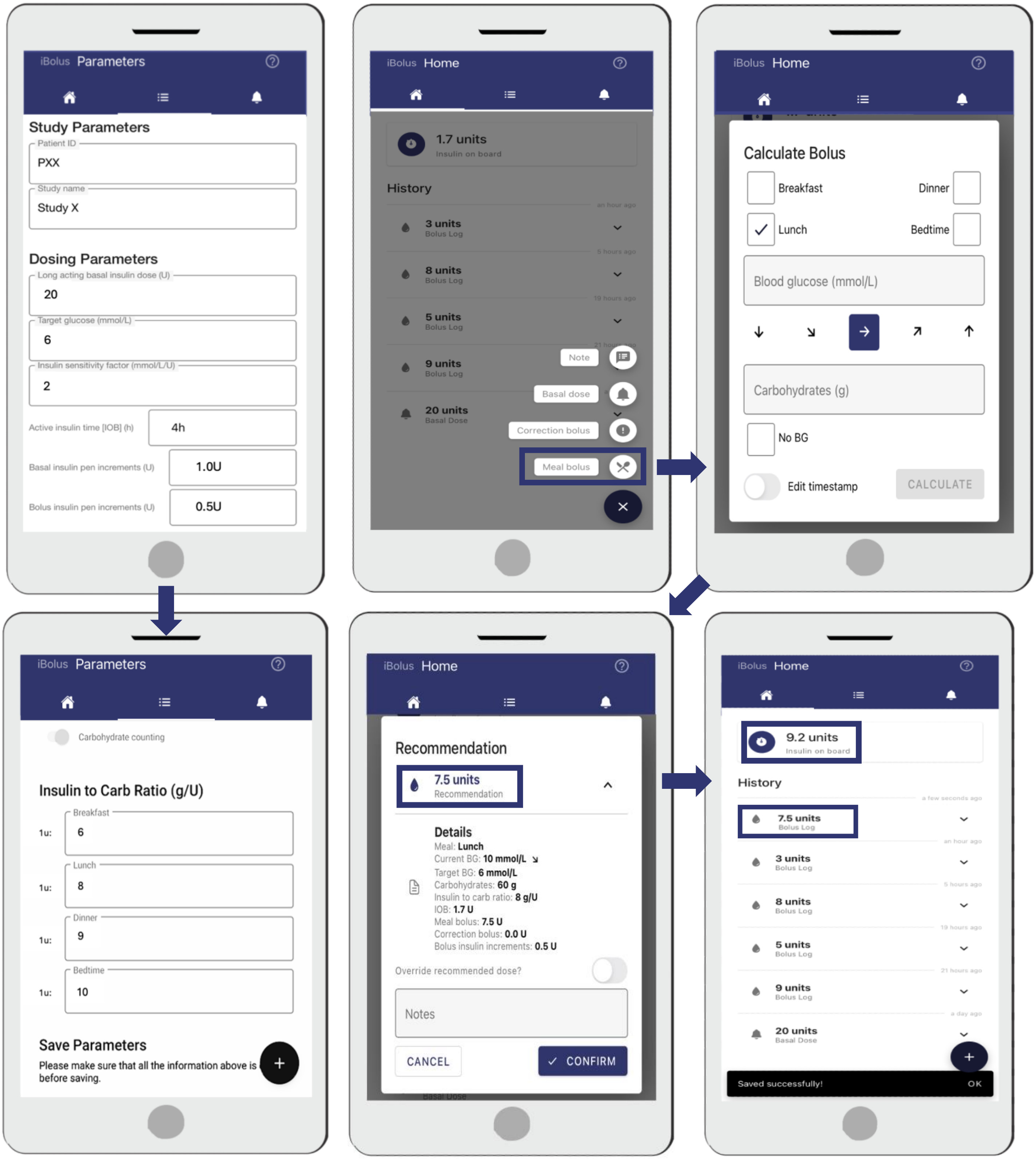

Supplement Fig. 5 | iBolus app user interface.
Screenshots of the iBolus app interface showing the parameters screen (top left, bottom left), meal bolus feature (top middle, top right), calculated meal bolus dose (bottom middle), and confirmed meal bolus with updated active insulin-on-board on main home screen (bottom right).
