# Supplementary material for: A Bayesian decision support system for automated insulin doses in adults with type 1 diabetes on multiple daily injections: a randomized controlled trial
Source: Nat Commun. 2025 Sep 29;16:8593. doi: 10.1038/s41467-025-63671-0 (PMC12479799; doi:10.1038/s41467-025-63671-0)
Supplement: Supplementary file 2 — Reporting summary [file 41467_2025_63671_MOESM2_ESM.pdf]

Corresponding author(s): Ahmad Haidar
Last updated by author(s): 8/22/2025

Reporting Summary

Nature Portfolio wishes to improve the reproducibility of the work that we publish. This form provides structure for consistency and transparency in reporting. For further information on Nature Portfolio policies, see our Editorial Policies and the Editorial Policy Checklist.

Statistics

For all statistical analyses, confirm that the following items are present in the figure legend, table legend, main text, or Methods section.

n/a	Confirmed	
☐	☒	The exact sample size (*n*) for each experimental group/condition, given as a discrete number and unit of measurement
☐	☒	A statement on whether measurements were taken from distinct samples or whether the same sample was measured repeatedly
☐	☒	The statistical test(s) used AND whether they are one- or two-sided<br>*Only common tests should be described solely by name; describe more complex techniques in the Methods section.*
☒	☐	A description of all covariates tested
☐	☒	A description of any assumptions or corrections, such as tests of normality and adjustment for multiple comparisons
☐	☒	A full description of the statistical parameters including central tendency (e.g. means) or other basic estimates (e.g. regression coefficient) AND variation (e.g. standard deviation) or associated estimates of uncertainty (e.g. confidence intervals)
☐	☒	For null hypothesis testing, the test statistic (e.g. *F*, *t*, *r*) with confidence intervals, effect sizes, degrees of freedom and *P* value noted<br>*Give P values as exact values whenever suitable.*
☒	☐	For Bayesian analysis, information on the choice of priors and Markov chain Monte Carlo settings
☒	☐	For hierarchical and complex designs, identification of the appropriate level for tests and full reporting of outcomes
☒	☐	Estimates of effect sizes (e.g. Cohen's *d*, Pearson's *r*), indicating how they were calculated

Our web collection on statistics for biologists contains articles on many of the points above.

Software and code

Policy information about availability of computer code

Data collection	The algorithm, developed in MATLAB R2018b, was executed weekly to provide recommendations with adjustments to basal and prandial insulin parameters throughout the study. As proprietary intellectual property, the algorithm code cannot be made publicly available. Glucose data were collected via the LibreView platform, while meal, insulin, and participant parameter data were entered through the iBolus app and securely transferred to a Google Firebase server.
Data analysis	Quantitative statistical analyses were performed using R v12.1 and MATLAB 2020. The NVivo 12 software was used to manage, store, and analyze the qualitative data.

For manuscripts utilizing custom algorithms or software that are central to the research but not yet described in published literature, software must be made available to editors and reviewers. We strongly encourage code deposition in a community repository (e.g. GitHub). See the Nature Portfolio guidelines for submitting code & software for further information.

Data

Policy information about availability of data

All manuscripts must include a data availability statement. This statement should provide the following information, where applicable:

- Accession codes, unique identifiers, or web links for publicly available datasets
- A description of any restrictions on data availability
- For clinical datasets or third party data, please ensure that the statement adheres to our policy

The study protocol is provided in the Supplementary Information. Raw data cannot be made publicly available due to restrictions in the informed consent form. However, deidentified individual participant data, including baseline characteristics and outcome measures (HbA1c, CGM and insulin metrics, and survey responses) are available upon request. Requests should be directed to the corresponding author by email. Data will be shared at no cost for non-commercial research purposes, subject to approval by the Research Ethics Board of the McGill University Health Center. Following approval, data will be transferred securely within three months. Data will become available three months after publication and will remain available for five years.

Research involving human participants, their data, or biological material

Policy information about studies with human participants or human data. See also policy information about sex, gender (identity/presentation), and sexual orientation and race, ethnicity and racism.

Reporting on sex and gender	In this study, there were 37 females and 47 males. We collected baseline data on gender; however, we did not conduct a sex- or gender-based sub-analysis.
Reporting on race, ethnicity, or other socially relevant groupings	We collected baseline data on race and ethnicity; however, we did not conduct a race- or ethnicity-based sub-analysis.
Population characteristics	Participant characteristics of adults with type 1 diabetes on multiple daily injections: overall mean age was 38 (12) years, diabetes duration was 22 (12) years, HbA1c was 8.6% (1.1), 44% were female, and 76% were regular sensor users (defined as at least three consecutive months of sensor wear prior to enrollment).
Recruitment	Participants were primarily recruited at the endocrinology clinic of the McGill University Health Centre. Written informed consent was obtained from all participants prior to initiating study procedures. Total study compensation was $300 CAD, paid proportionally in relation to completed study activities.
Ethics oversight	The study was reviewed and approved by the Research Ethics Board of the McGill University Health Centre. Authorization for the Jewish General Hospital to serve as a referral site was granted by the Convenance of the Centre intégré universitaire de santé et de services sociaux du Centre-Ouest-de-l'Île-de-Montréal.

Note that full information on the approval of the study protocol must also be provided in the manuscript.

Field-specific reporting

Please select the one below that is the best fit for your research. If you are not sure, read the appropriate sections before making your selection.

☒ Life sciences ☐ Behavioural & social sciences ☐ Ecological, evolutionary & environmental sciences

For a reference copy of the document with all sections, see nature.com/documents/nr-reporting-summary-flat.pdf

Life sciences study design

All studies must disclose on these points even when the disclosure is negative.

Sample size	We assumed a standard deviation of 0.8% for HbA1c, based on previous literature, and anticipated a between-group difference of 0.5%. As a result, we calculated that a sample size of 84 participants would provide 80% power at a two-sided significance level of 5%.
Data exclusions	No data was excluded from the analyses.
Replication	No replication of the findings was attempted, as this would require conducting a new clinical trial.
Randomization	Participants were randomized in a 1:1 ratio, stratified by prior sensor use, to receive either the McGill DSS (iBolus app with the optimization algorithm; experimental arm) or the non-adaptive iBolus app (control arm).
Blinding	No blinding was performed due to the nature of the intervention.

Reporting for specific materials, systems and methods

We require information from authors about some types of materials, experimental systems and methods used in many studies. Here, indicate whether each material, system or method listed is relevant to your study. If you are not sure if a list item applies to your research, read the appropriate section before selecting a response.

Materials & experimental systems

n/a	Involved in the study
☒	☐ Antibodies
☒	☐ Eukaryotic cell lines
☒	☐ Palaeontology and archaeology
☒	☐ Animals and other organisms
☐	☒ Clinical data
☒	☐ Dual use research of concern
☒	☐ Plants

Methods

n/a	Involved in the study
☒	☐ ChIP-seq
☒	☐ Flow cytometry
☒	☐ MRI-based neuroimaging

Clinical data

Policy information about clinical studies

All manuscripts should comply with the ICMJE guidelines for publication of clinical research and a completed CONSORT checklist must be included with all submissions.

Clinical trial registration	NCT04123054
Study protocol	The study protocol is provided in the Supplementary Information.
Data collection	Data collection took place at the McGill University Health Center, Royal Victoria Hospital, from March 5, 2020 through September 27, 2023.
Outcomes	The primary endpoint was the change in HbA1c from baseline to end of study. Secondary endpoints included total daily insulin dose and the percentage of time that sensor glucose levels were in the following ranges: 3.9-10 mmol/L, 3.9-7.8 mmol/L, <3.9 mmol/L, <3.0 mmol/L, >7.8 mmol/L, >10 mmol/L, >13.9 mmol/L, and >16.7 mmol/L. Additional metrics included mean sensor glucose and standard deviation. Sensor data were analyzed separately for three periods: overnight (23:00–07:00), daytime (07:00-23:00), and overall (24 hours).

Plants

Seed stocks	Not Applicable
Novel plant genotypes	Not Applicable
Authentication	Not Applicable
